# Identification of two KPC variants, KPC-204 and KPC-227, in ST11-K64 *Klebsiella pneumoniae* during prolonged hospitalization of a single patient

**DOI:** 10.3389/fmicb.2025.1543470

**Published:** 2025-06-17

**Authors:** Shijun Sun, Chaoe Zhou, Haijun Li, Liying Sun, Saiqi Qi, Xinmin Liu, Wanhai Wang

**Affiliations:** ^1^Department of Clinical Laboratory, The First Affiliated Hospital of Zhengzhou University, Zhengzhou, China; ^2^Department of Geriatrics, Peking University First Hospital, Beijing, China; ^3^Department of Clinical Laboratory, Peking University First Hospital, Beijing, China

**Keywords:** KPC variants, carbapenem resistance, ceftazidime-avibactam resistance, *Klebsiella pneumonia*, antimicrobial susceptibility

## Abstract

**Introduction:**

Carbapenem-resistant Klebsiella pneumoniae (CRKP) represents a critical global public health challenge due to its significant association with morbidity and mortality. Ceftazidime-avibactam (CZA) has emerged as an effective therapy against CRKP producing the serine carbapenemase KPC; however, resistance driven by novel KPC variants is increasingly reported.

**Methods:**

In this study, 27 CRKP isolates were collected from elderly pneumonia patients in China. Sequential isolates from a single patient undergoing prolonged hospitalization revealed dynamic resistance evolution.

**Results:**

Whole-genome sequencing identified KPC-227, a novel KPC variant, alongside the previously reported KPC-204. KPC-204, carrying a “DDK” insertion at position 270, conferred resistance to both carbapenems and CZA, while KPC-227, harboring a D179Y mutation, restored carbapenem susceptibility but maintained CZA resistance. Molecular docking analyses revealed that the D179Y mutation impaired meropenem hydrolysis by decreasing binding affinity. Additionally, colistin resistance was observed due to a novel mgrB mutation.

**Discussion:**

These findings highlight the high evolutionary potential of KPC enzymes and the importance of vigilance to curb the emergence and dissemination of resistance, which threatens the efficacy of critical lastresort antibiotics.

## Introduction

Carbapenem-resistant *Klebsiella pneumoniae* (CRKP) has emerged as a significant bacterial pathogen in recent years, posing a substantial threat to global public health. It disproportionately affects surgical patients, long-term hospitalized individuals, and those with compromised immune systems (Chen et al., 2014). In China, the clinical isolation rate of CRKP has risen from 6.4% in 2014 to 11.3% in 2021, underscoring that CRKP remains a significant multidrug-resistance (Wang et al., 2024). Currently, *Klebsiella pneumoniae* carbapenemase (KPC) is the most clinically significant serine carbapenemase globally, with over 80% of CRKP isolates in China carrying the KPC-2 enzyme (Hu et al., 2024). To address this challenge, ceftazidime-avibactam (CZA) has been developed as an effective treatment for carbapenem-resistant infections caused by KPC-producing strains (Karaiskos et al., 2021; Tumbarello et al., 2021). Clinical evidence from bloodstream infection studies demonstrates that CZA monotherapy significantly reduces mortality risk compared to traditional regimens, with adjusted odds ratios of 0.34 (95% CI 0.11–1.00) for in-hospital death and 0.18 (95% CI 0.04–0.77) for 30-day mortality, establishing it as a first-line therapeutic option (Boattini et al., 2023).

However, the widespread clinical use of ceftazidime-avibactam has been accompanied by the emergence of mutations in KPC enzymes, which compromise the clinical efficacy of avibactam. Emerging resistance to CZA due to KPC variants poses a growing threat to clinical outcomes, with treatment failures consistently associated with significantly higher mortality rates in recent clinical reports (Shields et al., 2017). This concern is substantiated by bloodstream infection cohorts revealing that CZA-resistant KPC-Kp infections carry substantial mortality burdens, demonstrating 22% in-hospital mortality and 16.2% 30-day mortality rates, particularly among patients with renal dysfunction or high comorbidity indices (Boattini et al., 2024a).

While ceftazidime-avibactam remains a cornerstone therapy against carbapenem-resistant Enterobacteriaceae, over 200 *bla*_KPC_ variants have been identified globally, 80% of which were characterized in the past three years (Ding et al., 2023). This accelerated evolution necessitates urgent molecular surveillance. Resistance to ceftazidime-avibactam has been linked to three mutational hotspots: (i) the Ω loop region (residues 164–179, which border the catalytic pocket), (ii) loop 237–243 (located between β3 and β4, adjacent to the conserved KTG motif), and (iii) loop 266–275 (situated between β5 and the α11 helices, some distance from the active site). Insertions and mutations near these hotspots may have direct effects on the enzyme’s structural dynamics and activity (Shields et al., 2017; Boattini et al., 2023; Ding et al., 2023; Boattini et al., 2024a).

This study reveals the alarming coexistence of two phenotypically distinct KPC variants (KPC-204 and KPC-227) isolated at sequential stages from a single long-term hospitalized patient, demonstrating adaptive mutational divergence under therapeutic pressure and underscoring the urgency for dynamic resistance profiling in CRKP management.

## Materials and methods

### Sample collection and isolation

A total of 27 non-repeated KPC-carrying *Klebsiella pneumoniae* isolates were collected from pneumonia patients with a history of ceftazidime-avibactam exposure in the geriatric department of a tertiary hospital between November 2022 and February 2023. All isolates were revived on Columbia blood agar plates at 35°C under aerobic conditions for 18–24 h and reconfirmed using matrix-assisted laser desorption ionization time-of-flight mass spectrometry (MALDI-TOF MS, Bruker Daltonik, Bremen, Germany) in the laboratory department of the First Affiliated Hospital of Zhengzhou University. *Escherichia coli* ATCC 25922 was utilized as a quality control strain for antimicrobial susceptibility testing. Additionally, *Escherichia coli* J53 (sodium azide-resistant), *Escherichia coli* DH5α, and *Klebsiella pneumoniae* ATCC 13883 were employed as recipient strains in conjugation and transformation experiments. The Ethics Committee of Peking University First Hospital, Beijing, China, provided ethical approval for this research (2022-yan-498). As it was an observational study, informed consent was waived.

### Antimicrobial susceptibility testing

The antimicrobial susceptibilities of the 27 isolates and their transconjugants were assessed using agar dilution (Mueller-Hinton agar, Oxoid, United Kingdom) and broth microdilution methods (Mueller-Hinton broth, BD Diagnostics, United States) (including tigecycline, colistin, and ceftazidime-avibactam) in accordance with the Clinical and Laboratory Standards Institute (CLSI) guidelines (M100 Ed35). Rationale for antibiotic panel selection: (i) Ceftazidime-avibactam (CZA) was prioritized as first-line agents against KPC-producers per IDSA (Infectious Diseases Society of America) recommendations; (ii) Colistin (COL) and tigecycline (TGC) represented salvage therapy options requiring resistance surveillance; (iii) Ceftazidime (CAZ) and aztreonam (ATM) served as mechanistic probes to exclude ESBL/MBL co-production; (iv) Levofloxacin (LVX) and amikacin (AMK) provided epidemiological data on fluoroquinolone/aminoglycoside resistance patterns in ST11 clones. All susceptibility tests were incubated at 35°C for 16–20 h. Quality control and minimum inhibitory concentration (MIC) results for all agents were interpreted based on CLSI breakpoints, except for tigecycline, which was evaluated using Food and Drug Administration (FDA) guidelines. The presence of *bla*_KPC_ was confirmed via PCR-based sequencing (primer KPC_F and primer KPC_R in Supplementary Table S1; Sun et al., 2024).

### Whole-genome sequencing and analysis

Genomic DNA of all the isolates and transconjugants were extracted using the TIANamp Bacteria DNA Kit (Tiangen Biotech, Beijing, China) followed by genomic DNA sequencing on the Illumina HiSeq X Ten platform (Illumina) using 150-bp paired-end reads with ≥ 100× coverage. Raw reads were quality-controlled using FastQC v0.11.9 and trimmed with Trimmomatic v0.39. Clinical isolates carrying *bla*_KPC–204_ and *bla*_KPC–227_ were simultaneously sequenced on the PacBio RS II system using P6/C4 chemistry, and 10-kb SMRTbell libraries were prepared following the manufacturer’s protocol (damage repair, end polishing, BluePippin size selection). Subreads were filtered through SMRT Link v5.0.1 (accuracy < 99% or read length < 5 kb were discarded). The FastQ data were assembled using Unicycler version 0.5.0 or SPAdes version 4.0.0, and the assembly was annotated using Prokka v1.14.6 (Bankevich et al., 2012; Wick et al., 2017; Seemann, 2068). Multilocus sequence types (STs), antimicrobial resistance genes, and plasmid replicon types were identified using Center for Genomic Epidemiology database,^1^ and all resistance genes were detected using Resfinder^2^ and Basic Local Alignment Search Tool (BLAST). Comparison of carbapenemase mutations of different KPC types using Beta-Lactamase DataBase (BLDB) database^3^ (Naas et al., 2017).

### Phylogenetic analysis

Multiple sequence alignments of whole-genome annotated GFF3 files from 27 isolates. Core genes were identified using Roary v3.12.0 with a 99% presence threshold (genes present in ≥ 99% of isolates) (Page et al., 2015). A concatenated alignment of 4,499 core genes (total length 4.1 Mb) was generated for phylogenetic reconstruction. An initial maximum-likelihood phylogenetic tree was constructed with RAxML v8.2.12 under the GTRGAMMA model (1,000 bootstrap replicates), and recombination events were identified and removed using ClonalFrameML with the RAxML-generated tree as input. The final clonal genealogy was inferred by re-running RAxML v8.2.12 on the recombination-filtered alignment (Stamatakis, 2014; Didelot and Wilson, 2015; Kozlov et al., 2019). The resulting tree was visualized using the Interactive Tree of Life (iTOL) v6.9.1 (Letunic and Bork, 2024).

### Gene cloning and functional analysis

To investigate the role of novel KPC variants in bacterial carbapenem resistance, the full-length open reading frames (ORFs) of different KPC variants were amplified by PCR (primer KPC_full_F and primer KPC_full_R in Supplementary Table S1) using PrimeSTAR Max DNA Polymerase (TaKaRa) in the Clinical Laboratory Department of the First Affiliated Hospital of Zhengzhou University. Purified PCR products (0.2 pmol) were incubated with 1 μL T-Vector pMD-19 (TaKaRa, a pUC19-derived cloning vector) and 5 μL DNA Ligation Mighty Mix (TaKaRa) at 16°C for 2 h. All PCR products were subjected to bidirectional Sanger sequencing by Tsingke Biotechnology (Beijing, China) using the primers listed in Supplementary Table S1. The recombinant plasmids pUC19-KPC-204 and pUC19-KPC-227 were transformed into competent *Escherichia coli* DH5α cells, incubated on ice for 30 min, and subjected to a 45-s heat shock at 42°C. The cells were immediately transferred to SOC medium (TaKaRa) and incubated at 37°C for 1 h, followed by plating onto LB agar plates (1.5% agar) containing 100 μg/mL ampicillin and incubation at 37°C overnight. The resulting colonies were verified by PCR (primer KPC_F and primer KPC_R in Supplementary Table S1) and subsequently tested for antimicrobial susceptibility using the broth microdilution method. The recombinant plasmids were extracted and electroporated into *Klebsiella pneumoniae* ATCC 13883.

### Plasmid transferability and stability

The transferability of the plasmids carrying *bla*_KPC–204_ and *bla*_KPC–227_, were determined by a conjugation assay on KP134, KP168 and azide-resistant *E. coli* J53 (using mid-log phase cultures (OD600 ≈ 0.5). Three independent biological replicates were performed, each including triplicate technical replicates (*n* = 3 membranes per biological replicate) The donor and recipient strains were mixed at a ratio of 1:3 on a sterile nitrocellulose membrane (0.22 μm pore size) placed on non-selective LB agar plates, followed by overnight incubation at 37°C as previously described (Sun et al., 2024). The transconjugants were selected on selected on China blue agar plates supplemented with 4 mg/L of ceftazidime and 150 mg/L of sodium azide. Transconjugants were confirmed by MALDI-TOF MS and PCR amplification of *bla*_KPC_ variants using specific primers. The transconjugants were confirmed by MALDI-TOF MS and PCR. Conjugation efficiency was calculated as the number of transconjugants per donor cell at time 0, using the formula: Efficiency (%) = (Transconjugants CFU/mL)/(Donors CFU/mL at t0) × 100. The initial donor cell density (t0) was determined by viable counting on LB agar containing 4 mg/L meropenem. Plasmid stability was assessed in three biological replicates, each with triplicate technical replicates (*n* = 3 cultures per replicate). Transconjugants were daily subcultured in non-selective LB broth (10 mL aliquots, 37°C with shaking at 200 rpm) for 7 consecutive days without antibiotic pressure. At 24-h intervals, bacterial suspensions were serially diluted and plated on non-selective LB agar. One hundred colonies per time point were replica-plated onto LB agar with or without 4 mg/L ceftazidime to determine the plasmid retention rate (%). Conjugation efficiency and plasmid stability experiments were determined as previously described (Sun et al., 2020). Full primer sequences and validation data are provided in Supplementary Table S1.

### Molecular docking

Molecular docking simulations were performed using AutoDock vina v1.1.2 software (Eberhardt et al., 2021). The three-dimensional protein structures of KPC-2 (PDB ID: 3RXX) was retrieved from the Protein Data Bank^4^ and selected for docking studies, as it provides a reliable representation of the native enzyme conformation while maintaining high resolution (1.8 Å) (Ke et al., 2012). For novel variants (KPC-227), three-dimensional models were generated using the web-based AlphaFold2 server^5^ with default parameters, followed by structural refinement through energy minimization using the AMBER22 force field (Jumper et al., 2021). Structural comparison between KPC-2 and its variants (KPC-204/KPC-227) was performed by aligning the predicted models to the reference KPC-2 structure (3RXX) using the “align” command in PyMOL v3.0 with default parameters. Root-mean-square deviation (RMSD) values were calculated to quantify backbone conformational changes. Chemical structures of meropenem and avibactam were obtained from PubChem. To prepare for docking calculations, the protein and ligand structures were converted into the PDBQT file format using the *prepare_ligand.py*^6^ and *prepare_protein.py*^7^ scripts in accordance with AutoDock protocols. Docking results were analyzed using PyMOL v3.0. Interactions between the ligands and protein active sites were visualized and evaluated based on binding affinities and conformations.

### Data availability

Complete sequences of the *bla*_KPC–227_ have been deposited with the GenBank databases under accession nos. PP770482. All sequencing data of isolates in this study were deposited in the NCBI genome database and organized under BioProject PRJNA595047.

## Results

### Characterization of total KPC-producing strains

Between November 2022 and February 2023, we collected carbapenem-resistant *Klebsiella pneumoniae* isolates from pneumonia patients (median age: 72 years; range: 65–89) with a history of ceftazidime-avibactam exposure in the geriatric ward of a tertiary hospital in northern China. A total of 27 CRKP isolates were obtained from 15 patients, all of whom had used ceftazidime-avibactam at least once in the previous month. Whole-genome sequencing (WGS) revealed that all isolates belonged to the ST11 clonal group (Table 1), with serotype distribution as follows: KL25 (1 isolate), KL47 (25.9%), and KL64 (70.4%), consistent with previous reports (Cai et al., 2024). The *bla*_KPC–2_ gene or its variants were detected in all isolates, confirming the dominance of KPC-mediated resistance in this cohort.

**TABLE 1:** Phenotype and genotype of 27 CRKP isolates in this study.

ID	MLST	K-tyke	KPC-type	Mutation of mgrB	Sample type	Patient ID	MIC (μg/mL)							
							**COL**	**CZA**	**TGC**	**MEM**	**LVX**	**AMK**	**CAZ**	**ATM**
KP164	ST11	KL64	KPC-2	WT	Blood	P9	0.5	4	1	16	8	64	256	64
KP165	ST11	KL64	KPC-2	WT	Sputum	P9	1	4	2	16	8	64	256	64
KP167	ST11	KL64	KPC-2	WT	Broncho-alveolar lavage	P9	0.5	4	1	16	8	64	256	64
KP168	ST11	KL64	KPC-227	G110T	Blood	P9	32	256	2	2	8	64	128	32
KP169	ST11	KL64	KPC-2	WT	Sputum	P9	0.5	4	1	16	8	64	256	64
KP170	ST11	KL64	KPC-227	G110T	Blood	P9	32	256	2	2	8	64	128	64
KP192	ST11	KL64	KPC-227	G110T	Skin	P9	32	256	2	2	8	64	256	64
KP291	ST11	KL64	KPC-227	G110T	Wound	P9	32	256	2	2	8	64	256	64
KP334	ST11	KL64	KPC-227	G110T	Wound	P9	32	256	2	2	8	64	256	64
KP337	ST11	KL64	KPC-227	G110T	Wound	P9	32	256	2	2	8	64	256	64
KP134	ST11	KL64	KPC-204	WT	Wound	P9	0.25	256	2	16	8	64	256	64
KP173	ST11	KL47	KPC-2	WT	Sputum	P10	1	4	2	16	16	1	256	64
KP174	ST11	KL47	KPC-2	Indel	Blood	P10	32	4	2	16	16	1	256	64
KP17	ST11	KL64	KPC-2	WT	Sputum	P1	0.5	4	2	16	16	64	256	64
KP18	ST11	KL64	KPC-2	Indel	Blood	P1	32	2	2	16	16	64	256	64
KP51	ST11	KL47	KPC-2	Indel	Sputum	P2	64	4	4	16	8	8	256	64
KP70	ST11	KL47	KPC-2	WT	Sputum	P3	0.5	2	16	16	8	64	256	64
KP75	ST11	KL47	KPC-2	WT	Broncho-alveolar lavage	P4	0.25	2	2	16	8	1	256	64
KP163	ST11	KL64	KPC-2	WT	Sputum	P5	1	4	1	16	8	64	256	64
KP90	ST11	KL64	KPC-2	WT	Sputum	P6	0.5	2	16	16	8	64	256	64
KP93	ST11	KL64	KPC-2	WT	Sputum	P7	0.5	2	16	16	8	64	256	64
KP124	ST11	KL25	KPC-2	WT	Sputum	P8	8	4	1	16	8	1	256	64
KP159	ST11	KL64	KPC-2	WT	Sputum	P11	2	4	4	16	32	64	256	64
KP154	ST11	KL64	KPC-2	WT	Sputum	P12	1	4	2	16	16	64	256	64
KP150	ST11	KL64	KPC-2	Indel	Sputum	P13	32	2	8	16	8	64	256	64
KP148	ST11	KL47	KPC-2	WT	Sputum	P14	1	4	2	16	8	4	256	64
KP137	ST11	KL47	KPC-2	WT	Sputum	P15	64	4	4	16	8	4	256	64

WT, Wild type. Antimicrobial agents are abbreviated as follows: COL, Colistin; CZA, ceftazidime-avibactam; TGC, Tigecycline; MEM, Meropenem; LVX, Levofloxacin; AMK, Amikacin; CAZ, Ceftazidime; ATM, Aztreonam.

### Sequential emergence of KPC variants in a single patient

A 68-year-old patient (P9) hospitalized for 59 days exhibited dynamic evolution of CRKP resistance. The initial pneumonia was attributed to CRKP based on: (1) high bacterial load in sputum cultures (> 10_5_ CFU/mL); (2) leukocytosis (WBC 15.2 × 10^9^/L) and elevated procalcitonin (PCT 2.8 ng/mL); and (3) lung CT showing bilateral lower lobe consolidations. Despite transient co-detection of Corynebacterium striatum (wound secretion) and Candida tropicalis (sputum) during the mid-infection phase, CRKP persistently dominated all subsequent microbiological profiles until the terminal stage. On the 11th day post-surgery (December 27, 2023), the first CRKP strain (KP165) was isolated from a sputum culture, and the strain was found to carry the *bla*_KPC–2_ gene. The treatment of P9 was subsequently switched to tigecycline, ceftazidime-avibactam, and a single-day course of polymyxin, which was discontinued due to an allergic reaction. Clinical resolution was achieved in the following week, evidenced by normalization of body temperature (36.8°C), decreased WBC (8.1 × 10^9^/L) and PCT (0.5 ng/mL). However, after 15 days of ceftazidime-avibactam treatment (January 26, 2023), a CRKP strain (KP168) was isolated from wound secretions. Susceptibility testing revealed resistance to ceftazidime-avibactam and a significant reduction in resistance to meropenem. Sequencing results indicated that this strain carried the *bla*_KPC–227_ gene.

**FIGURE 1:**
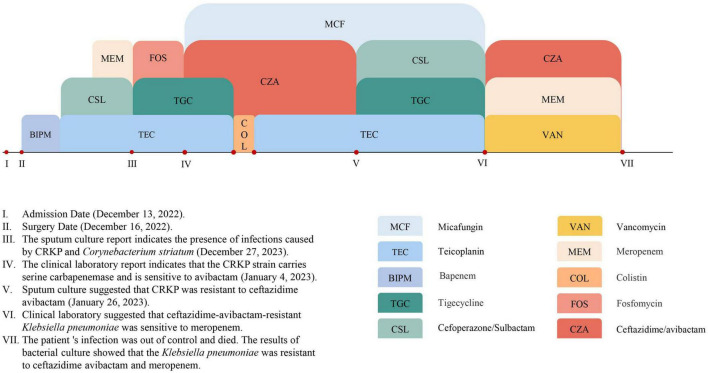
Timeline of major antimicrobial treatments administered to patient P9 during hospitalization. At time point V, the first isolate carrying *bla*_KPC–227_ was identified. At time point VII, the first isolate carrying *bla*_KPC–204_ was identified.

Clinically, tigecycline was administered for 2 weeks as a subsequent anti-infective treatment but proved ineffective. Based on laboratory reports indicating meropenem susceptibility, the treatment regimen was adjusted to a combination of ceftazidime-avibactam and meropenem. Initially, this combination effectively controlled the infection. However, after 2 weeks, the infection progressively worsened, ultimately leading to the death of P9. Before the patient passed away (February 8, 2023), a blood culture isolated CRKP (KP134) that was resistant to both meropenem and ceftazidime-avibactam. Sequencing identified another KPC mutant, *bla*_KPC–204_, in this strain. The primary antibiotic regimen for this patient is summarized in Figure 1.

### Identification of two KPC mutant genotypes and phenotypes

**FIGURE 2:**
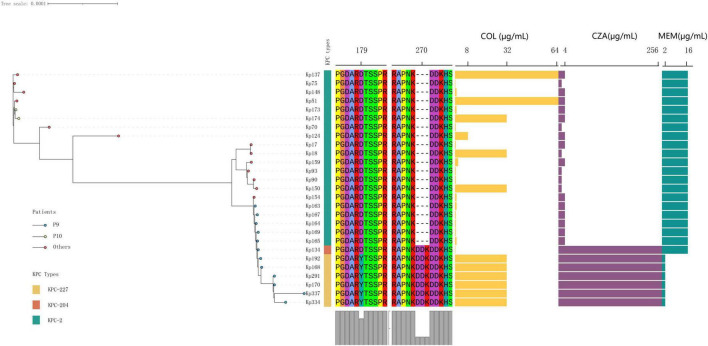
The phylogenetic relationships and antimicrobial susceptibility profiles of the collected isolates were analyzed. All isolates were identified as belonging to the ST11 *Klebsiella pneumoniae* clonal group. The orange, yellow, and green blocks in the first column correspond to KPC-204, KPC-227, and KPC-2, respectively. Blue endpoints denote isolates obtained from patient P9. Abbreviations for antimicrobial agents are as follows: COL (colistin), CZA (ceftazidime-avibactam), and MEM (meropenem).

Phylogenetic analysis revealed that all CRKP strains isolated from patient P9, each carrying different KPC genes, belonged to a single evolutionary clade (Figure 2). This finding suggests that, under the selective pressure of antibiotics during the patient’s 2-month hospitalization, the strain underwent resistance evolution. Two distinct KPC variants, designated *bla*_KPC–204_ and *bla*_KPC–227_, were isolated from strains KP134 and KP168, respectively. These genes were cloned into *Escherichia coli* and *Klebsiella pneumoniae* for functional characterization.

**FIGURE 3:**
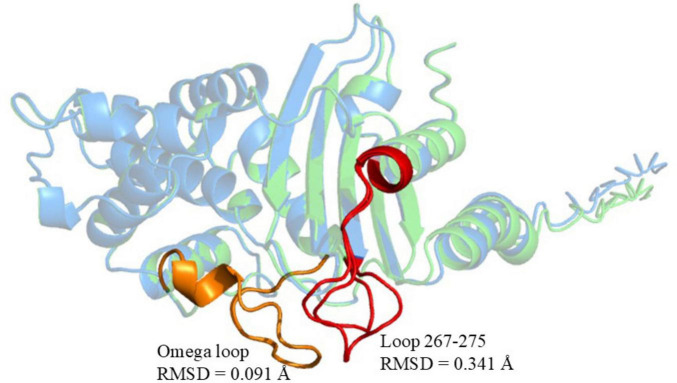
Structural comparison of KPC-227 (blue) and KPC-2 (green). Red regions: Conformational changes in KPC-227 (vs. KPC-2), including the distorted 267–275 loop (RMSD = 0.341 Å). Yellow regions: Conserved Ω-loop in KPC-2 (RMSD = 0.091 Å vs. KPC-227). Global structural conservation: Backbone alignment RMSD = 0.100 Å.

Compared to KPC-2, KPC-204 exhibited a “DDK” amino acid repeat at position 270 (in the loop 267–275 of KPC), a mutation already detected in other CRKP strains at the time of this writing, indicating the potential for its clinical spread and warranting close monitoring (Gong et al., 2024). In contrast, KPC-227 contains both the “DDK” insertion and an additional “D179Y” mutation at amino acid position 179 (in the Omega loop of KPC, Figure 3).

Gene cloning experiments demonstrated that strains carrying KPC-204 and KPC-227 exhibited significantly higher ceftazidime-avibactam MIC values (from 4/4 mg/L to ≥ 256/4 mg/L) compared to KPC-2-carrying controls, suggesting impaired avibactam inhibition efficacy. Notably, in *Klebsiella pneumoniae* ATCC 13883, the meropenem MIC for KPC-204-carrying strains was 8 mg/L, identical to that of KPC-2-producing strains, whereas the MIC for KPC-227 was reduced to 1 mg/L (Table 2). This indicates that while both variants confer resistance to ceftazidime-avibactam, KPC-204 retains significant carbapenemase activity comparable to KPC-2, whereas KPC-227 shows attenuated hydrolysis of carbapenems.

**TABLE 2:** Antimicrobial susceptibility profiles of the isolates carrying the *bla*_KPC_ and their transconjugants and transformants.

Bacteria	Description	KPC-type	MIC (μg/mL)		
			**MEM**	**CZA**	**CAZ**
KP134	Donor of *bla*_KPC–204_	KPC-204	16	256	128
KP134-T	Transconjugants of *bla*_KPC–204_	KPC-204	4	64	64
DH5α-*bla*_KPC–204_	Transformants of *bla*_KPC–204_	KPC-204	1	128	128
ATCC13883-*bla*_KPC–204_	Transformants of *bla*_KPC–204_	KPC-204	8	128	128
KP168	Donor of *bla*_KPC–227_	KPC-227	2	256	128
KP168-T	Transconjugants of *bla*_KPC–227_	KPC-227	1	64	64
DH5α-*bla*_KPC–227_	Transformants of *bla*_KPC–227_	KPC-227	0.25	32	64
ATCC13883-*bla*_KPC–227_	Transformants of *bla*_KPC–227_	KPC-227	1	32	64
KP165	Donor of *bla*_KPC–2_	KPC-2	16	4	256
KP165-T	Transconjugants of *bla*_KPC–2_	KPC-2	4	2	64
DH5α-*bla*_KPC–2_	Transformants of *bla*_KPC–2_	KPC-2	2	2	64
ATCC13883-*bla*_KPC–2_	Transformants of *bla*_KPC–2_	KPC-2	8	2	128
*E.coli* J53	Recipient for conjugation	–	0.032	0.125	1
*E.coli* DH5α	Recipient for transformantion	–	0.032	0.125	1
*K. pneumoniae* ATCC13883	Recipient for transformantion	–	0.064	0.125	1

Antimicrobial agents are abbreviated as follows: MEM, Meropenem; CZA, ceftazidime-avibactam; CAZ, Ceftazidime.

**FIGURE 4:**
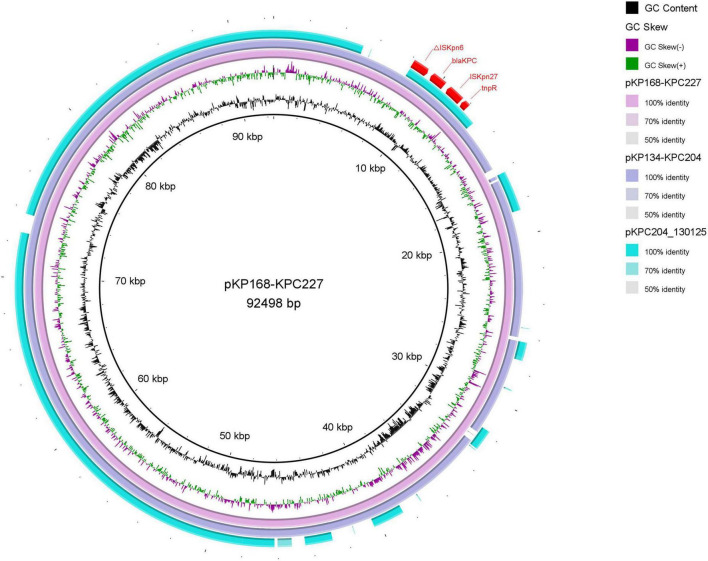
Comparison of plasmid structures between *bla*_KPC–204_ and *bla*_KPC–227_. Using the plasmid carrying *bla*_KPC–227_ as a reference, the plasmid structure of *bla*_KPC–204_ was found to be identical to that of *bla*_KPC–227_ during the treatment period in patient P9. The outermost red region represents the genetic environment surrounding the KPC mutants, with no significant differences compared to previously reported structures.

Sequencing results revealed that *bla*_KPC–227_ is located on an IncFII plasmid (92,498 bp) within a *Tn*4401 transposon context. Comparative analysis revealed that *bla*_KPC–227_, *bla*_KPC–204_, and the previously reported pKPC204_130125 all resided within conserved *Tn*4401 transposon contexts on IncFII plasmids (Figure 4). Specifically, *bla*_KPC–227_ exhibited > 99% structural identity with *bla*_KPC–227_ in our cohort, while both shared identical backbone organizations with pKPC204_130125 (Gong et al., 2024), strongly supporting clonal dissemination of these *bla*_KPC_ harboring plasmids across distinct clinical strains. Additionally, the plasmid encoded multiple resistance genes, including aminoglycoside resistance genes [*aph(3’)-IIa*, *rmtB*, *aadA8b*], beta-lactam resistance genes (*bla*_*CTX*–*M*–15_, *bla*_*TEM*–1B_), and sulfonamide resistance genes (*sul1*, *dfrA12*). Furthermore, all strains isolated from patient P9 harbored a 215,814 bp virulence plasmid carrying four virulence genes: *rmpA (regulator of mucoid phenotype associated with hypervirulence), rmpA2 (homologous virulence enhancer), iucABCD (aerobactin siderophore biosynthesis cluster), and iutA (aerobactin receptor).* These genes collectively confer hypermucoviscosity phenotype and enhance iron acquisition capacity, which are hallmark features of hypervirulent *Klebsiella pneumoniae*.

### Assessment of plasmid transferability carrying *bla*_KPC–204_ and *bla*_KPC–227_

Conjugation experiments and plasmid stability assays were conducted to assess the transferability of pKP168_KPC227, pKP134_KPC204, and pKP165_KPC2. All plasmids carrying KPC mutants were successfully transferred into *E. coli* J53 (Table 2), with average transfer frequencies of (1.9 ± 0.8) × 10^−5^, (3.4 ± 0.7) × 10^−5^, and (3.0 ± 0.5) × 10^−5^ (*n* = 3 biological replicates), respectively, without statistically significant differences observed (Kruskal-Wallis test (non-parametric ANOVA equivalent). Additionally, after 7 consecutive days of daily subculturing, plasmid retention rates in KP168, KP134, and KP165, plasmid retention rates were 94% (941/1,000 CFUs), 90% (898/1,000 CFUs), and 87% (874/1,000 CFUs), respectively, indicating stable maintenance even in the absence of antibiotic selection pressure.

### Emergence and characterization of colistin-resistant mutants

Despite patient P9 receiving only a brief colistin treatment due to an allergic reaction, high-level colistin-resistant strains (KP168 and KP170) were detected shortly afterward. Sequencing revealed a G110T point mutation in the *mgrB* gene of these colistin-resistant strains, resulting in a valine-to-glycine substitution at position 37. This mutation had not been previously reported. Functional validation using CRISPR gene-editing technology confirmed that the in situ G110T mutation in the *mgrB* gene significantly increased colistin resistance in the host strains (Supplementary Table S2).

### Molecular docking

**FIGURE 5:**
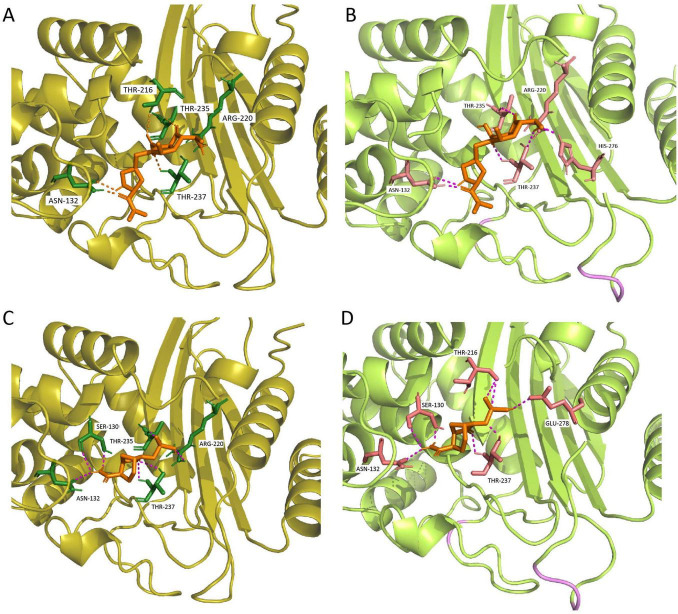
Molecular docking results for KPC-227 and KPC-2 with meropenem and avibactam. **(A)** Docking results of KPC-2 with meropenem. **(B)** Docking results of KPC-227 with meropenem: Compared to KPC-2, KPC-227 lacks the hydrogen bond at THR216 but gains a hydrogen bond at HIS276. **(C)** Docking results of KPC-2 with avibactam. **(D)** Docking results of KPC-227 with avibactam: KPC-227 lacks hydrogen bonds at THR235 and ARG220 but gains hydrogen bonds at GLU278 and THR216.

Compared to KPC-2, the mutations in KPC-227 are located in the Omega loop (residues 164–179) and the loop spanning residues 267–275. Significant structural differences were observed in the 265–275 loop region, where an insertion of three amino acids (DDK) at position 270 resulted in an expansion of the 267–275 loop (Figure 3). Molecular docking simulations were performed to analyze and illustrate the interactions of KPC-227 and KPC-2 with avibactam and meropenem, respectively (Figure 5).

KPC-227 is unable to form a side-chain hydrogen bond with THR216 in its interaction with meropenem. Additionally, the binding free energy of KPC-227 with meropenem (−5.9 kcal/mol) was notably higher (less negative) than that of KPC-2 (−7.5 kcal/mol), which may explain the reduced affinity of KPC-227 for meropenem. For avibactam, KPC-227 lacks the hydrogen bond formed with THR235 in KPC-2, and its binding free energy is also significantly reduced (−7.1 kcal/mol vs. −6.03 kcal/mol). These findings explain why KPC-227 confers resistance to ceftazidime-avibactam while losing hydrolytic activity against meropenem.

## Discussion

Infections caused by CRKP impose a significant economic burden and are associated with mortality rates two to three times higher than those of carbapenem-sensitive strains (Ding et al., 2023). The primary mechanism of CRKP resistance to carbapenems is the production of carbapenemases, with KPC-2 being the most prevalent. Avibactam acts by covalent acylation of the β-lactamase target in KPC-2. Since its approval in China (2019), ceftazidime-avibactam (CZA) has been considered among the most effective agents for treating infections caused by KPC-producing strains, particularly *Klebsiella pneumoniae* (Yin et al., 2019). However, resistance to CZA due to KPC mutations has emerged rapidly following its widespread clinical use (Li et al., 2024; Oueslati et al., 2019). Since 2019, reports of *bla*_KPC_ variants have increased sharply, and as of November 20, 2024, 229 KPC variants have been cataloged in the NCBI Reference Sequence (RefSeq) database (NCBI Pathogens RefGene Database).

Among KPC-2 mutations, the Ω loop and the amino acid loop spanning residues 267–275 are recognized as hotspots for ceftazidime-avibactam resistance (Hobson et al., 2022; Shen et al., 2022; Ding et al., 2023; Sun et al., 2023; Gong et al., 2024). Mutants conferring resistance to both ceftazidime-avibactam and meropenem remain rare; While dual resistance to ceftazidime-avibactam and meropenem remains uncommon, emerging evidence reveals broader mutational landscapes. Beyond the 267–275 loop mutations (KPC-29, KPC-154, KPC-204) (Arcari et al., 2023; Gong et al., 2024), deletions in the β3-β4 loop (e.g., Δ242-GT-243 in KPC-14) have also been reported to confer cross-resistance, underscoring the structural plasticity of KPC variants in evading β-lactam/β-lactamase inhibitor combinations (Boattini et al., 2024b). Intriguingly, the Ω-loop mutant KPC-227 exemplifies an opposite evolutionary trajectory: its D179Y mutation is predicted to disrupt the Asp179-Thr235 hydrogen bond (Figure 5C), based on AlphaFold2 structural modeling, thereby destabilizing the Ω-loop conformation. This structural may perturbation not only abolishes meropenem hydrolysis by impairing substrate binding but also paradoxically enhances ceftazidime diffusion through channel widening, while reducing avibactam inhibitory efficiency (Alsenani et al., 2022). Experimental validation of this mechanism is warranted to confirm the predicted hydrogen bond disruption. These observations collectively underscore the dual role of the Ω loop—its structural integrity is critical for carbapenemase activity, yet targeted modifications in this region may simultaneously drive resistance to novel inhibitors.

In this study, we characterized the co-occurrence of KPC-227 (a novel variant) and KPC-204 (previously described Gong et al. (2024)) within the same patient, revealing their divergent evolutionary trajectories under therapeutic pressure. Our findings align with prior reports of therapeutic failure in Ω-loop mutant infections: even when meropenem susceptibility is restored (e.g., KPC-227), heteroresistant subpopulations (e.g., KPC-204) may persist, leading to rapid resistance reversion under monotherapy pressure (Gaibani et al., 2018; Bianco et al., 2020). This phenomenon underscores the risk of relying solely on meropenem for ceftazidime-avibactam-resistant but carbapenem-susceptible strains. Combinatorial regimens (e.g., meropenem/vaborbactam or imipenem/relebactam) may provide broader coverage by targeting both KPC variants and potential AmpC/ESBL co-producers (Corcione et al., 2023).

Importantly, Ω-loop mutants like KPC-227 pose unique diagnostic challenges: their attenuated carbapenemase activity may evade phenotypic carbapenemase tests (e.g., mCIM), potentially misclassifying them as ESBL producers and prompting inappropriate carbapenem use (Bianco et al., 2022b). To mitigate this risk, molecular surveillance (e.g., *bla*_KPC_ variant PCR) and selective media for ceftazidime-avibactam resistance should be prioritized in endemic regions. Failure to detect such strains not only compromises treatment but also neglects infection control measures, enabling silent outbreaks (Bianco et al., 2022a).

Notably, while this study focused on KPC-mediated resistance mechanisms, we observed concurrent resistance evolution in patient P9. Despite minimal colistin exposure (discontinued due to allergy), strains developed a novel *mgrB* mutation (V37G) associated with high-level colistin resistance (MIC = 32 mg/L). Notably, the rapid emergence of the G110T mgrB variant (V37G) under limited colistin exposure highlights the potential for unexpected resistance selection through collateral evolutionary pressures. These findings highlight the remarkable adaptability of resistance genes in *Klebsiella pneumoniae* and emphasize the importance of vigilant clinical monitoring and timely detection of resistance. Effective laboratory surveillance and resistance profiling are essential for guiding antimicrobial therapy, improving patient outcomes, and mitigating the impact of antimicrobial resistance.

## Data Availability

Complete sequences of the *bla*_KPC_ have been deposited with the GenBank databases under accession nos. PP770482. All sequencing data of isolates in this study were deposited in the NCBI genome database and organized under BioProject PRJNA595047.

## Footnotes

1
https://genomicepidemiology.org/services/

2
http://genepi.food.dtu.dk/resfinder

3
http://www.bldb.eu/

4
https://www.rcsb.org/

5
https://colab.research.google.com/github/sokrypton/ColabFold/blob/main/AlphaFold2.ipynb

6
https://github.com/MunibaFaiza/Virtual-Screening_files/blob/main/prepare_ligand.py

7
https://github.com/Martin-Floor/prepare_proteins
