# Supplementary material for: Identification of two KPC variants, KPC-204 and KPC-227, in ST11-K64 Klebsiella pneumoniae during prolonged hospitalization of a single patient
Source: Front Microbiol. 2025 Jun 17;16:1543470. doi: 10.3389/fmicb.2025.1543470 (PMC12209373; doi:10.3389/fmicb.2025.1543470)
Supplement: Supplementary file 1 [file Table_1.docx]

Table S1

Primers used in this study

| **Primer** | **Product** | **Sequence (5’–3’)** | **Annealing temperature (◦C)** |
| --- | --- | --- | --- |
| KPC_F | Fragment of *bla_KPC_* | GGCGGCTCCATCGGTGTGTA | 60 |
| KPC_R |  | AATTGGCGGCGGCGTTATCA |  |
| MgrB_F | Fragment includes the *mgrB* | GAAGGCCGTGCTATCCTGG | 61 |
| MgrB_R |  | ACAACAGACCGACAAGCAGC |  |
| KPC_full_F | Fragment includes the *mgrB* | AGCTCCACCTTCAACAAAGG | 58 |
| KPC_full_R |  | TGTGACAGTGGTTGGTAATCC |  |

The product of primers KPC_F & KPC_R: AATTGGCGGCGGCGTTATCACTGTATTGCACGGCGGCCGCGGACAGCTCCGCCACCGTCATGCCTGTTGTCAGATATTTTTCCGAGATGGGTGACCACGGGAACCAGCGCATTTTTGCCGTAACGGATGGGTGTGTCCAGCAAGCCGGCCTGCTGCTGGCTGCGAGCCAGCACAGCGGCAGCAAGAAAGCCCTTGAATGAGCTGCACAGTGGGAAGCGCTCCTCAGCGCGGTAACTTACAGTTGCGCCTGAGCCGGTATCCATCGCGTACACACCGATGGAGCCGCC

The product of primers MgrB_F & MgrB_R: GAAGGCCGTGCTATCCTGGCGACATTGCGTACTGATGCGGAGAGTGGAGTGAAAAAATTACGGTGGGTTTTACTGATAGTCATCATAGCAGGCTGCCTGTTGCTGTGGACTCAGATGCTTAACGTAATGTGCGACCAGGATGTTCAGTTTTTCAGCGTCATTTGCACTATTAATAAATTTATTCCGTGGTAAGACATTTTTCTGCCGACTGATTTCCTTCTGCGCCGCGGGTGGTAGAATGAACGCCTTCTCTTTGAGGTGGTGAAATGAGTGAGTTACTAAATCCTGGGATTTTAAATCTGGCATCGCTGGCTGTGTCCGTGGCGCTGCTGCTTGT

The product of primers KPC_full_F & KPC_full_R: TGTGACAGTGGTTGGTAATCCATGCCGCCCGCAAGGGCGGCGGTGGTGGGCCAATAGATGATTTTCAGAGCCTTACTGCCCGTTGACGCCCAATCCCTCGAGCGCGAGTCTAGCCGCAGCGGCGATGACGGCCTCGCTGTGCTTGTGATCCTTGTGATCCTTGTTAGGCGCCCGGGTGTAGACGGCCAACAACAATAGGTGCGCGCCCAGTGGGCCAGACGACGGCATAGTCATTTGCCGTGCCATACACTCCGCAGGTTCCGGTTTTGTCTCCGACTGCCCAGTCTGCCGGCACCGCCGCGCGGATGCGGTGGTTGCCGGTCGTGTTTCCCTTTAGCCAATCAACAAACTGCTGCCGCTGCTGGCGCAGCCAGTGCTGAGCCCAGTGTTCAGTTTTTGTAAGCTTTCCGTCACGGCCGCGGCGATGAGGTATAGCGCGCATTCGCCTGGGATGGCGGAGTTCAGCTCCAGCTCCCAGCGGTCCAGACGGAAACGTGGTATCGCCGATAGAGCGCATGAAGGCCGTCAGCCCGGCCGGGCCGCCCAACTCCTTCAGCAACAAATTGGCGGCGGCGTTATCACTGTATTGCACGGCGGCCGCGGACAGCTCCGCCACCGTCATGCCTGTTGTCAGATATTTTTCCGAGATGGGTGACCACGGGAACCAGCGCATTTTTGCCGTAACGGATGGGTGTGTCCAGCAAGCCGGCCTGCTGCTGGCTGCGAGCCAGCACAGCGGTAGCAAGAAAGCCCTTGAATGAGCTGCACAGTGGGAAGCGCTCCTCAGCGCGGTAACTTACAGTTGCGCCTGAGCCGGTATCCATCGCGTACACACCGATGGAGCCGCCAAAGTCCTGTTCCGAGTTTAGCGAATGGTTCCGCGACGAGGTTGGTCAGCGCGGTGGCAGAAAAGCCAGCCAGCGGCCATGAGAGACAAGACAGCAGAACTAGACGGTGATACAGTGACATCAACGATATTCCTTGTTTGAAGGTGGAGCT

Table S2

Functional validation of the T110G point mutation in *mgrB*

| Bacteria | Description | MIC (μg/mL) |
| --- | --- | --- |
| KP168 | Colistin-resistant *Klebsiella pneumoniae* isolated from patients | 32 |
| KP168_mgrB_T110G | Restoration of mgrB point mutation in KP168 to wild type using CRISPR-cas9 | 0.5 |
